# Strategies for relapse prevention among people with schizophrenia in KwaZulu-Natal Province, South Africa: Healthcare providers’ perspectives

**DOI:** 10.1371/journal.pone.0316313

**Published:** 2025-03-10

**Authors:** Joyce Protas Mlay, Thirusha Naidu, Suvira Ramlall, Krushika Patankar, Khadija Israel, Laura Esquivel, Laura Curran, Sbusisiwe Sandra Mhlungu, Makhosazane Zondi, Richard Lessells, Andrew Tomita, Jennifer I. Manuel

**Affiliations:** 1 Discipline of Public Health Medicine, School of Nursing and Public Health, University of KwaZulu-Natal, Durban, South Africa; 2 Health Economics and HIV and AIDS Research Division (HEARD), University of KwaZulu-Natal, Durban, South Africa; 3 Department of Innovation in Medical Education, Faculty of Medicine, University of Ottawa, Ottawa, Canada; 4 Department of Psychiatry, University of KwaZulu-Natal, Durban, South Africa; 5 Department of Public Health and Primary Care, University of Cambridge, Cambridge, United Kingdom; 6 Silver School of Social Work, New York University, New York, New York, United States of America; 7 University of South Florida, Tampa, Florida, United States of America; 8 Private Practice, Durban, South Africa; 9 Centre for the AIDS Programme of Research in South Africa (CAPRISA), Durban, South Africa; 10 Discipline of Psychology, School of Applied Human Sciences, University of KwaZulu-Natal, Durban, South Africa; 11 KwaZulu-Natal Research Innovation and Sequencing Platform (KRISP), College of Health Sciences, University of KwaZulu-Natal, Durban, South Africa; 12 Centre for Rural Health, School of Nursing and Public Health, University of KwaZulu-Natal, Durban, South Africa; 13 School of Social Work, University of Connecticut, Hartford, Connecticut, United States of America; University of Pretoria, SOUTH AFRICA

## Abstract

**Introduction:**

Relapse is a significant challenge among people with schizophrenia and is broadly recognized by the aggravation of positive or negative symptoms, the need for re-hospitalization, more intensive case management, and/or changes in medication. The quality of inpatient care and proper transition to outpatient care are crucial in reducing the risk of relapse. Healthcare providers play vital roles in ensuring the continuity of care after patients are discharged from the hospital. Little is known about the roles of preventing relapse from the perspective of healthcare providers. This study explored the currently existing strategies for preventing relapse from the perspective of healthcare providers.

**Methods:**

We captured the view of healthcare providers providing services to psychiatric patients using a qualitative methodological approach with descriptive phenomenology. We conducted audio-recorded, in-depth interviews with 15 consenting clinical providers from a public psychiatric hospital in Durban, South Africa. To facilitate analysis, we used Dedoose software (SocioCultural Research Consultants, LLC [www.dedoose.com]), and the themes were inducted from the data.

**Results:**

Six major themes inducted from the analysis: Preparing patients and caregivers for discharge; Developing consistent and caring therapeutic relationships; Using an active approach to transition; Working with patients and caregivers concurrently; Creating and sustaining interagency connections; and Facilitating alternative forms of treatment.

**Conclusions:**

Discharge planning and preparation are needed to ensure smooth transitions from hospital to outpatient care for relapse prevention. The healthcare system should ensure the availability of human resources for health at all levels of health facilities, and multidisciplinary teamwork will help a successful transition.

## Introduction

Relapse in schizophrenia is broadly recognized by the aggravation of positive or negative symptoms, the need for re-hospitalization, more intensive case management, and/or changes in medication [1]. Most individuals with schizophrenia experience relapse at some point during their illness [2,3]. The contributory factors for relapse include poor adherence to anti-psychotic medication, substance use, and lack of insight [4]. Relapse following inpatient psychiatric treatment is a significant problem among individuals with serious mental illness (e.g., schizophrenia and schizoaffective disorder) [5]. In South Africa (SA), the primary data collected directly from facilities in 2017 reveal that the average national readmission rate for mental health inpatients is 24.2% over three months. Most inpatient and outpatient costs are attributed to care at specialized psychiatric hospitals [6]. In rural SA, KwaZulu-Natal Province (KZN), a retrospective analysis of longitudinal hospital admission data between January 2011 and February 2015 has found a relapse rate of approximately 50% within the first year after leaving inpatient psychiatric treatment [4]. The strategies for preventing relapse among schizophrenia comprise the quality of inpatient and outpatient care, especially primary health care, and the ability to ensure continuity of care after discharge [7,8]. The period immediately after being discharged from the hospital is a critical time, as it represents a heightened risk for hospital readmission, suicide, and interruptions in the continuity of care for patients [4,9]. In randomised controlled trials, the critical time intervention model (CTI) and assertive community treatment (ACT) have effectively prevented homelessness and re-hospitalization among patients with severe mental illness after discharge to the community settings [10–12]. CTI model provides time-limited, intensive support during critical transitions to ensure stability and connect individuals to community resources, while ACT offers ongoing, comprehensive, community-based psychiatric care and support for individuals with severe mental illness. Considering the high cost of implementing these models, ensuring the coordination of inpatient and outpatient care is highly recommended [13]. The quality of inpatient and outpatient care, as well as the effectiveness of the mental health system in providing well-coordinated care and support during a patient’s transition from hospital to ongoing community-based care, is crucial [2]. Review studies indicate that approximately half of the patients with schizophrenia and related disorders miss their first scheduled outpatient appointment after discharge [14,15].

Healthcare providers play a critical role during discharge planning and preparation to ensure the continuity of care [9,16]. The quality of inpatient psychiatric care may reduce the risk of relapse by preparing the patients with coping mechanisms and ensuring adherence to treatment after discharge [15]. Studies suggest poor treatment adherence and re-hospitalization are due to low patient knowledge of the importance of pharmacological management; however, implementing an appropriate plan before discharge may facilitate patient transition [8,15]. Good clinical practice in mental health care would consider at least one scheduled meeting with all parties concerned before discharge and family involvement as core elements [14]. There are several advantages to discharge planning, including an increase in patient knowledge, a decline in clinical symptoms, and a reduction in hospital admission frequency [2].

In SA, discharge planning and referral to aftercare support are severely lacking during psychiatric hospitalization, increasing the likelihood of relapse and recidivism and the risk of death, as evidenced by the Life Esidimeni tragedy discharge. In this tragedy, people with mental illnesses were placed at step-down facilities that were not accredited or registered to provide mental health care without proper assessment for ongoing care support, resulting in a severe humanitarian crisis [17]. Also, mental health services-related support is inadequate outside specialized public sector psychiatric hospital settings [4]. In SA, a cross-sectional empirical study evaluating the National Mental Health Policy Framework and Strategic Plan (2013–2020) found that inpatient care is the predominant form of treatment. The study highlighted the need to develop outpatient care services and emphasized that reducing admission rates could lead to significant cost savings [6]. There is a dearth of literature about the strategies for preventing relapse from the perspective of healthcare providers in SA. This study aims to address these gaps in the literature by exploring the current strategies for relapse prevention following discharge from inpatient psychiatric care from the perspectives of healthcare providers.

## Methods

### Study design and setting

This study was conducted at a public/government-specialized psychiatry hospital in Durban, SA. The hospital has a psychiatric unit and provides outpatient and inpatient care for patients diagnosed with mental illness, including schizophrenia. The hospital has 930 beds, including 130 specialized psychiatric beds and 400 district hospital beds, XDR Provincial TB 320 beds, and Orthopaedic Spinal 80 beds. The district hospital does the 72 hours of psychiatry admission and assists psychiatry’s medical comorbidities/emergencies, and specialised psychiatry deals with psychiatry admissions that require specialised care. Patients’ average length of stay ranges from 14 days to 18 months, largely due to community and residential care challenges. Over 90% of admissions are involuntary, “under Section 36 of the Mental Health Care Act No.17 of 2002” [18]; the hospital sees an average of 900 outpatients monthly. The hospital is the referral service for Ethekwini, with a population of about 3.5 million, and the Ilembe district. The qualitative approach using descriptive phenomenology entailed the use of audio-recorded face-to-face interviews with healthcare providers involved in providing services to psychiatric patients.

### Participants: sampling and recruitment

Participants included 15 consenting healthcare providers from a public psychiatric hospital in KZN, SA. We used reputational case selection to construct a purposive sample of clinical practitioners who best represent those providing services to psychiatric patients [19]. Providers were eligible to participate if they met the following criteria: 1) above 18 years of age, 2) English or isiZulu speaking, and 3) be a clinical staff member who has direct contact with patients and works in psychiatric treatment, including psychiatrists, nurses, psychologists, social workers, medical practitioner, and nursing operational managers. A researcher/interviewer verbally explained the study to potential participants and obtained written informed consent. JM, an experienced researcher in qualitative methods, trained the interviewer on qualitative methods, data management, and participant safety protocols.

### Data collection

A trained researcher/interviewer with an honor degree in psychology and fluent in English and IsiZulu conducted audio-recorded face-to-face interviews with healthcare provider participants. An interview guide was developed and included open-ended questions about participants’ views on discharge planning and preparation, returning to the community, access to and use of family and social supports, community resources, and outpatient services; challenges related to medication adherence; and potential leverage points and intervention strategies to address service/support gaps. Study participants received an honorarium of R50 [$3.50] to cover meal costs. The interviews lasted approximately 60 minutes, and the data was collected from 10th April 2019 to 24th July 2019. To ensure credibility, the interviewer conducted member checking during data collection, explored the phenomenon using varied questioning, and undertook daily transcriptions to inform the following interviews. In addition, confirmability was ensured by keeping interview notes, audio recording the interviews, and doing verbatim transcriptions, which enabled quotes to be presented. Lastly, reflexivity was observed by the interviewer, who reflected on the data collection process, kept daily notes, and did not interrupt the participants but sought clarity when necessary. Also, the phenomena were described during the analysis/analyses, and the manifest, not latent, meaning from the data was presented.

### Data analysis

The researcher/interviewer transcribed the recording, and the senior researcher (JM) trained four additional data analysts (KP, KI, LE, LC) on a stepped analytic approach to examining the data. To ensure dependability, all five analysts read all transcripts and coded text relevant to the broad study research questions. Each analyst developed initial codes using a first-level process of constant comparison within and between interview transcripts, ultimately developing individual codes. Analysts met to discuss their sets of initial and grouped codes, comparing and contrasting codes and quotations among analysts, providing a second-level constant comparison; this process led to an initial codebook. Three of the five analysts conducted a third stage of constant comparison to derive the salient themes emerging from the data. Discrepancies were resolved through discussion until a consensus was reached among three analysts. We used Dedoose software (SocioCultural Research Consultants, LLC [www.dedoose.com]) to facilitate analysis.

### Ethics

Ethical approval was obtained from the University of KwaZulu-Natal Biomedical Research Ethics Committee (#BREC: BFC 189/17). The permission for data collection was obtained from the KwaZulu Natal Department of Health (KZN-DoH) Province and hospital management, and all study participants received copies of their written informed consent. To ensure confidentiality, participants were identified by number and not by name throughout the data collection and analysis process.

## Results

### Sample

Providers represented a number of different roles at the hospital, including nurses (n =  5), psychiatrists or psychiatrists in training (n =  4), occupational therapists (n =  2), social worker (n =  1), clinical psychologist (n =  1), medical practitioner (n =  1), and nursing operational manager (n =  1). The majority of the providers were female, primarily in their 50s, and most were psychiatric nurses.

We identified six major themes: preparing patients and caregivers for discharge, developing consistent and caring therapeutic relationships, using an active approach to transition, working with patients and caregivers concurrently, creating and sustaining interagency connections, and facilitating alternative forms of treatment.

#### Theme 1. Preparing patients and caregivers for discharge.

A prominent theme from the analysis was discharge preparation for patients and caregivers. Discharge preparation refers to all activities and actions that point toward treatment design leading to discharge planning and preparation for reintegration into the community. This theme consists of four subthemes: medication adherence, skills building, readiness for change, and providing education to increase knowledge and reduce stigma.

**Medication adherence.** Most healthcare providers agreed that poor anti-psychotic medication adherence is a significant problem for outpatients but not so much in the hospital ward. Therefore, they insisted on assisting the patient in continuing with follow-up on the hospital schedule after discharge. They explained various mechanisms used to support the patients and caregivers to continue with care after discharge consisting of psychoeducation, shifting responsibility, multidisciplinary involvements, and needs for community health services:

**Psychoeducation:** Most healthcare workers emphasize improving the diagnosis and medication knowledge to improve adherence after discharge. They claimed they are using the predischarge group session and psychoeducation to improve the understanding of the importance of drug adherence.

*“We explain to them why they must take the medication and the possible side effects because some are scared that the side effects will permanently affect them. We explain to them the positive things about taking medication.* ***” Staff #15, Professional Nurse***

**Shifting responsibility to family:** Healthcare providers mentioned that when the patients are institutionalized in the ward, they easily follow the ward’s routine and schedule. Also, the nurses practice direct treatment observation in the ward to ensure medication adherence; the family’s involvement is crucial to actively supervising after discharge to improve adherence.

*“If we identify an issue with adherence, we do direct observation to ensure that the patient is taking medication.”* ***Staff #2, Nursing Operational Manager***

*“I also educate the person and their family and stressed the importance of supervision. Explain that the aim is not to drug the patient but to make them more functional. If all else fails, we opt for injectable and involve the family because mental illness usually affects the whole family, not just one person.”* ***Staff #13, Psychiatry Registrar***

*“It’s how patients view medication, their attitude, and family. If a family doesn’t take an active role, it becomes a problem because some of these patients are heavily dependent on their parents, and once they lose their parents, they stop taking the medication, and some end up on the street.”* ***Staff #7, Professional Nurse***

**Multidisciplinary involvement:** Healthcare providers mentioned that patients’ needs are specific and require teamwork in teaching, especially drug adherence. Also, insisting on involving the family in the group session to enhance the overall therapeutic experience, fosters a supportive environment, and promotes a more comprehensive understanding of the patient’s needs and challenges:

*Also, involve the team, the nurses, the social worker, and the occupational therapist. We all try to develop insight into this patient and involve the family. Involvement of the Family is also important, so having them on board helps a lot. Multiple strategies can be used to improve adherence.”* ***Staff #8, Senior Psychiatrist***

*“We offer a full rehabilitation program with psychology and occupational therapy services. We also counsel them on medication compliance and try to include the family even though we are short-staffed.”* ***Staff #7, Professional Nurse***

**Need for community health services:** The healthcare worker suggested the need for health services in the community, e.g., home visits to prepare the environment for support, including the availability of the medicine at the clinics and direct tracking to make sure the patients follow up:

*“The lack of community health services and someone who can follow up on the patient. If the patient can get support in the first month of being discharged, it could make a difference, but if they get lost in the gaps in that first month, we will lose the patient until the next relapse. So if we can get a system to monitor the patient after discharge, it would make a big difference”.* ***Staff #8, Senior Psychiatrist***

*“Hospital, I think the processes are there to assist patients. The issue is in the community and the patient’s home, but otherwise, in the past, there was a program where the patients would be visited by professional nurses (Home visits). It wasn’t particularly the duty of the hospital per se. It’s more of the community level, so for me, its revival and restoring such structures would be beneficial”.* ***Staff #2 Nursing Operational Manager***

**Skills building.** Healthcare providers explained that the level of function of the patients varies after the number of relapses; therefore, they are aiming at providing the clients with skills to carry out daily activities, especially after discharge, because, in the ward, the patients are not allowed to sleep during the day. Skills-building varies from basic daily hygiene and social skills to vocational skills.

*“Yes, we do psychoeducation, like the activity clock. Teaching them time management. Most are not working because they are on disability grants, so they have 8 hours daily doing nothing. So we help them plan their day and possible activities to keep them occupied. Because they are not allowed to sleep here at the hospital during the day, we are helping them manage their time outside the hospital”.* ***Staff #6, Occupational Therapist***

*“When you look at my ward, it’s a high risk, and most are low functioning. So we teach them basic hygiene skills, such as bathing and cleaning. Social skills include making friends, having a good relationship with their family, and getting along in the ward with others. I also teach them how to budget and spend their grant money. We cover a lot of issues in the psychoeducation program. “* ***Staff #7, Professional Nurse***

*“There is this patient that lives right by the hospital. I taught him life skills and self-care. Today, he is self-employed and takes good care of himself* ***”. Staff #7, Professional Nurse***

*“I do the groups, planned activities, worksheets, discussion for goals, and practical stuff. We redo our flyers to make them more current and eligible. I plan the groups and facilitate them. We do life skills such as cooking and sewing”* ***.Staff #5, Occupational Therapist***

**Readiness for change.** Healthcare providers mentioned that, sometimes, patients fail to adhere to the follow-up and interrupt medication due to a lack of insight and not accepting their illness. Providing patients with education helps them change and follow up on the instructions.

*“Poor insight or poor understanding of the condition. A patient comes to the ward with poor understanding, telling you he won’t take medication because he is not sick. So, for us to promote adherence, you need to promote understanding; if the understanding is poor, the adherence will also be poor. These things go hand in hand. Once the pt understands that he is sick and needs medication, he will start taking the medication accordingly”.* ***Staff# 1. Professional Nurse***

*“Sometimes it works a miracle because you find that even a patient that has been reluctant will be the first on the line to come to get medication. I found that it’s not just one thing that creates that magic. Continuous enforcement is one concern because if I identify that a patient needs constant motivation, what happens when they are out of the hospital”.* ***Staff #2, Nursing Operational Manager***

**Providing education to increase knowledge and reduce stigma.** Refers to all activities and actions that aim at helping the patients to live in their community affected by stigma and discrimination. Healthcare providers mentioned that stigma affects healthcare utilization by the patients and the family: most of them prefer not to use the clinics near where they live because of stigma and discrimination. Lack of transport money to adhere to the follow-up visit to the distant clinic also brings challenges; therefore, knowledge provision on how to respond to any language associated with the stigma is very important.

*“Educating them on how to behave in the community and how to deal with the stigma”* ***Staff # 4, Social Worker***

*“Firstly, the stigma issue is not just talking about stigma but orientating the public at large, having open days in the hospital, and showing them what we do in the wards. Show them the seclusion room, rooms with burglar gates, and explain why we have them because when they see that, all they think about is jail”.* ***Staff #7, Professional Nurse***

Also, healthcare providers mentioned that, at times, patients lose their jobs because of their history of being admitted to the psychiatry department; therefore, the occupational therapist works hand in hand with the patients to return to their job station.

*“Oh ja, I can think of one. It’s a lady who worked as a manager; she was admitted for psychosis. While she was admitted, she had problems at work, and her boss could not understand why she had to be in the hospital for so long, so they wanted to employ someone else. So, the occupational therapist had to step in and intervene by writing letters to her boss and doing a functional assessment to prove that she could still return to work. So, our team fought to get her job back. So, more was done, and it turned out well because she was back at work. Fully functional”.* ***Staff #15, Professional Nurse***

#### Theme 2. Developing consistent and caring therapeutic relationships.

This theme refers to the patient’s/client’s ability to trust the provider when receiving services. It also refers to the service provider’s behavior that displays trust building. Healthcare providers mentioned that during inpatient care, they ensure therapeutic relationships to help build trust with their patients. Once the patients and family trust them, it is easy to follow what has been instructed; one said:

*“Medication adherence is a huge problem. I think doctors work on this rational model that if a person is sick and I prescribe medication, they will take it, not realizing that many factors are involved, like the doctor-patient relationship, which will determine if the patient will take it. And I think doctors are confused about why patients don’t take their medication. If you don’t trust your health professional, you are unlikely to listen to them.”* ***Staff #11, Clinical Psychologist***

*“Creating a trusting relationship is important if you are a mental health care practitioner. First, I find that the more the patient trusts the person they are interacting with, the more they open up about their issues, leading to speedy and better recovery* ***.” Staff #2, Nursing Operational Manager***

*“I think patients very often feel like they are not heard, and by sitting down and talking to them about a particular medication, why they have to be on it, and making them aware that we know you have side effects, I think it makes them feel more supported.”* ***Staff #3, Psychiatrist***

#### Theme 3. Using an active approach to transition.

This refers to all activities that aim to create an environment for the patients and families that ensures a successful transition. This theme encompassed four sub-themes: assessing needs, connecting the patients with the services, resource provision, and outreach and tracking.

**Assessing needs.** Healthcare providers insisted that before discharge, they usually assess the patient’s needs for accommodation and help the patient accordingly, e.g., if the patient doesn’t have a place to live, the social worker will find a home:

*“Then we send the patient to the family. If there is nowhere to go, that is the social worker’s duty to place the patient in a home”.* ***Staff #1, Professional Nurse***

Additionally, the healthcare providers mentioned that they assess the need for a disability grant and assist patients with the necessary arrangements before discharge:

*“If the patient needs a disability grant or family support, we will try to address that while the patient is still in the ward,”* ***Staff #3, Psychiatrist***

**Connecting the patients with the service.** Healthcare providers usually assess patients’ needs during hospital care and connect them to available services. If the patient is unemployed and doesn’t have an income, the doctor will refer the patient to the social worker, and the patient will be assisted with the grant application one said:

*‘The first support is health education that we offer for patients. If a patient is unemployed and doesn’t have an income, the doctor will refer the patient to the social worker, and the social worker will give the patient Sassa forms to apply for the grant”.* ***Staff #1, Professional Nurse***

Also, before discharge, the social worker assesses whether the patient needs counseling and refers them to the nearest clinic if necessary.

*“Those who need counseling, I see them and also refer them to their nearest clinic.”* ***Staff # 4, Social Worker***

**Resource provision**. Healthcare providers claim that patients are engaged in various services during hospital care and continue to access them even after discharge. Therefore, before being discharged, the patients and caregivers are informed about these services:

*“I also try and do a lot of family intervention because they also go through the steps of illness. They need reassurance. You need to make them aware of the services available and build and empower them with knowledge about the patient’s condition.”* ***Staff #10, Registered Nurse***

*“Sometimes they need a referral to a social worker, psychologist, and occupational therapist. And then, even post-discharge, we make appointments for patients to attend those services. But the social worker offers various services, from grants to arranging placements”.* ***Staff #2, Nursing Operational Manager***

**Outreach and tracking.** Healthcare providers claimed that during admission, they request the patient’s physical address and the relative’s number and give them to the outpatient department staff for tracking and ensuring connection after discharge.

*“On admission, we try to get as many contact numbers as possible and their address, so even if they live in a rural area, we try to get the landmark at least. So it’s never been a problem”.* ***Staff #15. Professional Nurse***

*“If I see a patient not coming to the clinic, I call the family to find out about the patient. But I can’t do it for all the patients. It’s just the ones referred to me”.* ***Staff # 4, Social Worker***

Also, healthcare providers suggested that there is a need for community services, e.g., home visits as they were doing in the past, and a system that will allow monitoring and see if the patients continue with care:

*“Hospital wise, I think the processes are there to assist patients. The issue is in the community and the patient’s home, but otherwise, in the past, there was a program where the patients would be visited by professional nurses (Home visits). It wasn’t particularly the duty of in hospital per se; it’s more of the community level, so for me, its revival and restoring such structures would be beneficial”.* ***Staff #2, Nursing Operational Manager***

*If the health department had one system of registering patients, we would be able to see if a patient is attending a clinic in KZN so they don’t abuse medication and collect from different clinics. They even sell the treatment to drug abusers. So this system would save the department a lot of money.* ***Staff #4***, ***Social Worker***

*There is no system in place. We are just doing the basics. The systems and policies are not being practiced or put into place.* ***Staff #7***, ***Professional Nurse***

#### Theme 4. Working with patients and caregivers concurrently.

Healthcare providers insisted that throughout the treatment process, they engage family members to help the patients continue with the treatment; also, they insisted on the need for training to help them perform the task well:

*“We offer a full rehabilitation program with psychology and occupational therapy services. We also counsel them on medication compliance and try to include the family even though we are short-staffed.”* ***Staff #7, Professional Nurse***

*“Maybe get families more involved. Maybe have a meeting with the family and the multidisciplinary team. Involve the family in the patient’s treatment process.”* ***Staff #15, Professional Nurse***

*“The nursing staff also need a lot of training in psychoeducation patients. You can’t just talk generally to psych patients; you need to create talks suited for each patient’s needs”.* ***Staff #7, Professional Nurse***

#### Theme 5. Creating and sustaining interagency connections.

In this theme, healthcare providers mentioned that for most patients, there’s a gap between them being in the hospital and going back home; there is not enough service in between this gap, though a few community agencies have been created to help the patients in the community.

*“In the community, there are no structures to support patients and their families. Before, we had nurses that did home visits, especially visiting patients who relapsed.”* ***Staff #4, Social Worker***

*“Back home, in an ideal world, they should be discharged to a community with a program to support them. But they are discharged back home, and it’s not a conducive environment”.* ***Staff #11, Clinical Psychologist***

*“I developed an extensive database of community agencies, plus we formed an advocacy group including mental health practitioners, so we have done a lot to establish a community of mental health organizations.”* ***Staff #11, Clinical Psychologist***

#### Theme 6. Facilitating alternative forms of treatment.

In this theme, healthcare providers mentioned that other forms of treatment should be strengthened, e.g., Social support and counseling from a psychologist are essential, as some patients may not meet the criteria for starting medication treatment. The psychologist can help with counseling and help the patients.

*“So I think like social support, and that impacts mental health. They might not have enough symptoms to meet diagnostic criteria, so in a way, our role is blurred. People need psychologists, but what a psychologist can offer is not exactly what they need”.* ***Staff #11, Clinical Psychologist***

Healthcare providers insist on the importance of occupational therapists, who engage with patients before discharge and help to explain the importance of preventing relapse.

*“OT has a predischarge plan. We do it on Monday till Wednesday at 2 pm, and we take them through relapse prevention, healthy eating plans, coping skills, goal setting, time management, and spirituality. Sr. XXX does Thursday. We ensure by the end of the week, we have notes on the observations that were noted in groups. Psychology also has a predischarge group once a week”.* ***Staff #5, Occupational Therapist***

Lastly, the health care provider suggested that patients with a history of substance abuse should be discharged to the rehab center, not home, to avoid continuing with drugs.

*“We need to gear up and make it known that if a patient is asymptomatic and abusing substances, they are referred to Rehab. You rarely find a patient discharged from us going to rehab”.* ***Staff # 10, Registered Nurse***

## Discussion

The current study explored the current strategies for relapse prevention from the perspective of healthcare providers in SA. The themes inducted focused primarily on concrete individual and interpersonal strategies that providers can implement to support a transition from the hospital among patients with schizophrenia. We provide a discussion for each of the major themes below.

### Preparing patients and caregivers for discharge

Most studies show that relapse commonly occurs shortly after hospitalization due to failure to engage with care after discharge [20,21]. In this study, healthcare providers reported using predischarge planning to prepare the patients and caregivers to continue with care and prevent relapse. While the effectiveness of predischarge planning in relapse prevention is well established [2,8,14], novel findings from the current study detail specific elements for healthcare providers incorporated during discharge planning to ensure patients continue with care. These elements include emphasizing medication adherence, skills building, readiness for change, and providing education to increase knowledge and reduce stigma.

Notably, the healthcare providers reported using psychoeducation to educate the patients about their medication regimen, side effects, and any anticipated challenges that make them responsible for their health and help improve treatment adherence. The importance of psychoeducation in treating mental health patients has been well-reported [11]. Furthermore, healthcare providers recognized that stigma significantly impacts medication adherence. Patients may choose to visit a distant clinic out of fear of discrimination, while lack of transport money can contribute to disengagement from care. To address these issues, the healthcare providers offered guidance on how patients can respond to stigmatizing language and educate them on strategies to mitigate the effects of stigma. Studies suggest poor treatment adherence and re-hospitalization are due to insufficient patient knowledge [22,23]. Effective mental health management requires a comprehensive approach encompassing psychological and pharmacological interventions.

Also, healthcare providers transfer responsibility by involving family members and caregivers in the patient’s care roles. They ensure that these individuals understand the patient’s treatment and actively engage them in providing support to enhance adherence. Good discharge practice in mental health care would consider at least one scheduled meeting with all parties concerned before discharge and family involvement as core elements [9,24–26]. The discussion of changing roles after discharge should also involve clinicians working in the outpatient departments; this is essential to ensure a successful transition. Moreover, healthcare providers emphasize using a comprehensive care team to identify patient and caregiver needs and employ active follow-up strategies in the community after hospital discharge to help improve medication adherence. Community structure should ensure close follow-up with the patients, including a phone call to remind the schedule and ongoing teaching to improve adherence [8,9,14]. This is also supported by the WHO Mental Health Action Plan 2013–2030 for Low and middle-income countries (LMICs), which enhances comprehensive, integrative, and responsive mental health and social care services in community–based settings [27].

The healthcare provider prioritizes skill development during discharge preparation, offering social skills training (SST) that includes comprehensive instruction in essential and life skills. This approach aims to support patients in reacquiring their functional abilities to enhance their quality of life [28,29]. Other studies show that the SST resulted in reduced length of hospital stay, reduced relapse, and improved social adaptive functions [2,30]. The occupational therapist should prioritize in-hospital training, provide comprehensive instruction, and facilitate skill development to assist patients in achieving improved function; the skills building should also include cognitive behavioral therapy. In addition to skills building, the healthcare provider also focuses on helping patients transform their attitudes towards medication and preparing them for post-discharge medication adherence. Regularly reinforcing positive attitudes towards treatment for patients and their family members becomes crucial in improving medication adherence and long-term clinical and functional outcomes.

### Developing consistent and caring therapeutic relationships

Consistent with existing research, our findings point to the importance of therapeutic relationships to help build trust with their patients [15,31]. The positive impact of the therapeutic relationship is associated with improved medication compliance, easier transition to community care, and improved outpatient treatment compliance [32–34]. Developing trust in some patients may be difficult; therefore, healthcare providers should be trained to develop a therapeutic relationship during treatment. This is also supported by The National Mental Health Policy Forum and Strategic Plan 2023 to 2030, which aim to improve access to quality mental health services and address mental health gaps [35].

### Using an active approach to transition

In this study, healthcare providers express their dedication to actively supporting patients in maintaining their care, which includes assisting with disability grant applications, which help reduce the financial barrier to healthcare utilization. Moreover, research has shown that financial difficulties pose a risk for relapse, as some patients may struggle to attend follow-up visits [2,11,36,37]. Furthermore, healthcare providers stated that before discharge, they make the patients aware of available resources (occupational therapy, social workers, and rehabilitation services) and reassure them that they can continue using the service even after release. Healthcare providers recommend discharging patients with substance use issues to a rehabilitation center instead of sending them home to prevent relapse. They also suggest the implementation of community health nursing services for close patient follow-up, in line with research findings [2]

### Working with patients and caregivers concurrently

In this study, healthcare providers highlighted their practice by involving family members through discharge sessions to help them understand their role in supporting patients after discharge. Engaging with the patient’s family enhances accessibility to care, provides essential support, and helps diminish societal stigmas surrounding the patient’s condition, which aligns with [24]. The engagement of family members with primary care providers can take various forms, ranging from basic functions like psychoeducation and addressing family needs to more specialized interventions, such as family assessment and family therapy. Family unity plays a critical role in the care of patients with mental illness, aligned with [26], which suggests that families are expected to be responsible for the care of such patients.

### Creating and sustaining interagency connections

In this study, healthcare providers claimed to use a low-intensity approach for follow-up to ensure effective connection after discharge by requesting patients’ phone numbers and sharing them with outpatient staff; this aligns with [38]. However, the use of critical time interventions (CTI) and assertive community treatment (ACT) have shown to be effective in outreach and tracking patients, especially those at high risk of relapse [11,12,20,31]. In Africa, setting community-based intervention that trains community health workers in primary community mental health support by providing education counseling and referrals to formal mental health services is highly recommended to ensure a successful transition. Community care workers play a pivotal role in monitoring and supporting psychiatric patients, and they serve as a bridge between them and the healthcare system [39]

### Facilitating alternative forms of treatment

In this study, healthcare providers emphasized the importance of comprehensive patient treatment. They stressed that a holistic approach to healthcare, including a range of treatment modalities, is essential for achieving the best possible patient outcomes. Studies show successful treatment and relapse prevention require a collaborative approach involving social workers, psychologists, and rehabilitation centers [28,40]. They also insisted that it is good to discharge the patients who abuse alcohol to rehabilitation centers rather than allowing them to return to the same environment because it increases the risks of relapse. The healthcare system should strengthen all treatments, including pharmacotherapy, psycho-social therapy, cognitive behavioral therapy, group therapy, family therapy, and occupational therapy. However, the healthcare providers did not provide guidance on the significance of traditional healers and religious beliefs. Increasing awareness and education about the UN Convention on the Rights of Persons with Disabilities (UNCRPD) and the SA White Paper on the Rights of Persons with Disabilities emphasize the importance of accommodating and supporting individuals within their social and cultural contexts.

### Study limitations

This study uses in-depth interviews to explore the perspectives of healthcare providers from a single hospital; the information given by healthcare providers might not necessarily reflect their actual roles and actions in clinical settings or those of other healthcare providers. Further study could use other methods, such as direct or indirect observations of practice, which will inform more about what is happening in the clinical settings and also should incorporate community health workers.

### Future directions

This study explores healthcare workers’ perspectives on the strategies for relapse prevention. Discharge planning and preparation are needed to ensure smooth transitions from hospital to outpatient care. Healthcare management should ensure the availability of sufficient human resources for healthcare provision at mental health facilities to allow a successful transition. More research is needed to assess the key factors contributing to readmission, and the government must also allocate resources to mental health and general health facilities to ensure the integration of mental health services.
